# Characterization of serum protein electrophoresis patterns and C-reactive protein in canine tick-borne diseases

**DOI:** 10.14202/vetworld.2021.2150-2154

**Published:** 2021-08-21

**Authors:** Sariya Asawakarn, Piyanan Taweethavonsawat

**Affiliations:** 1 Biochemistry Unit, Department of Veterinary Physiology, Faculty of Veterinary Science, Chulalongkorn University, Bangkok 10330, Thailand; 2 Biomarkers in Animals Parasitology Research Group, Chulalongkorn University, Bangkok 10330, Thailand; 3 Parasitology Unit, Department of Veterinary Pathology, Faculty of Veterinary Science, Chulalongkorn University, Bangkok, 10330, Thailand

**Keywords:** blood parasites, C-reactive protein, dog, electrophoresis, serum protein

## Abstract

**Background and Aim:**

Canine tick-borne diseases are important diseases with a worldwide distribution. In Thailand, the most important canine tick-borne diseases are ehrlichiosis, babesiosis, and hepatozoonosis. This study aimed to determine the serum protein electrophoresis patterns (SPEPs) and C-reactive protein (CRP) levels associated with *Ehrlichia canis***,** *Babesia canis*, or *Hepatozoon canis* single infections.

**Materials and Methods:**

A total of 650 canine blood samples were collected from animal hospitals and clinics in Bangkok and its vicinity to examine health status and blood parasite infection. Suspected blood parasite infections were examined by buffy coat thin blood smear and confirmed by polymerase chain reaction. Normal dog and positive *E. canis***,** *B. canis*, and *H. canis* single infections and serum protein profiles were determined by agarose gel electrophoresis. CRP concentration was measured by fluorescent immunoassay.

**Results:**

In dogs infected with *E. canis*, *B. canis*, and *H. canis* single infections, albumin levels and A/G ratios significantly decreased, whereas β2-globulin levels increased (p<0.05). The γ-globulin level significantly increased in *E. canis* and *H. canis* infections (p<0.05). A monoclonal gammopathy pattern wasi observed in *E. canis* and *B. canis* sngle infections, whereas β-γ bridging patterns and increased β- and γ-globulin fractions were found in *H. canis* single infections. The CRP level increased in dogs with blood parasite single infections and may be related to the pathogenesis of the infection.

**Conclusion:**

SPEPs and CRP levels can be used to monitor health status and blood parasite problems in infected dogs.

## Introduction

Canine tick-borne diseases are transmitted by hard tick species, such as the brown dog tick (*Rhipicephalus sanguineus*), which is common in Thailand [1] and is distributed worldwide [2]. Ticks not only feed on blood cells but also transmit different types of pathogens, such as protozoa, viruses, rickettsia, and bacteria [3], causing both morbidity and mortality. *Ehrlichia canis*, *Babesia canis vogeli*, and *Hepatozoon canis* are commonly found in Thailand [4,5]. Ehrlichiosis and babesiosis can develop when a dog is bitten by an infected tick, but hepatozoonosis is caused by its ingestion. *B. canis* subspecies *vogeli* is the main species causing canine babesiosis in Thailand [5]. *B. canis* is the large form of *Babesia* spp. *Babesia* organisms enter and multiply in the host erythrocytes. Clinical manifestations are anorexia, lethargy, pale mucous membranes, fever, jaundice, chronic nephropathy, and glomerulonephritis [6]. The severity of infection depends on the subspecies of canine babesiosis. *B. canis* causes a subclinical to a mild degree of infection [6,7]. *E. canis* is a Gram-negative intracellular rickettsia that can infect monocytes and lymphocytes in dogs [8]. Canine ehrlichiosis can be classified into three stages according to clinical signs: Acute, subclinical, and chronic. In the acute stage, clinical signs appear at 1-3 weeks after infection and include fever, weakness, lethargy, depression, lack of appetite, and limb edema. In the subclinical stage, the organism may be present for months to years without clinical symptoms. In the chronic stage, the infected dog has abnormal bleeding due to thrombocytopenia, severe weight loss, fever, difficulty in breathing due to lung inflammation, joint pain, seizures in some cases, lack of coordination, anemia, and kidney failure [9]. *H. canis* is an apicomplexan parasite that belongs to the family Hepatozoidae. The clinical signs of *H. canis* infection can vary from subclinical to severe and life-threatening. The most frequently observed clinical signs are anemia, extreme lethargy, intermittent fever, and emaciation [7].

Serum protein electrophoresis patterns (SPEPs) show fractions of two major types of protein: Albumin and globulin. Albumin is the single most abundant protein in the serum and is synthesized by the liver. Globulins are also synthesized by the liver, except for immunoglobulins. In dogs, globulin fractions can be separated into five fractions: α1, α2, β1, β2, and γ. The measurement of serum protein may help detect and monitor various diseases and pathological processes. SPEPs can be used as a diagnostic tool in a wide spectrum of diseases, including infectious and inflammatory diseases, renal, hepatic, and gastrointestinal disorders, immunodeficiency status, and paraproteinemia, caused by plasma cell neoplasia [10]. The acute-phase response is considered part of the innate host defense system, and the systemic effects include leukocytosis, fever, and increased blood cortisol. C-reactive protein (CRP) is one of the acute-phase proteins and is synthesized by hepatocytes, smooth muscle cells, macrophages, endothelial cells, lymphocytes, and adipocytes. It is a major acute-phase protein in dogs and is part of the γ-globulin fraction. Its concentration increases dramatically in response to inflammation, infection from pathogens (including bacteria and parasites), and injury and has been used as a predictive marker for disease risk and to monitor the response to treatment [11].

This study aimed to investigate the SPEPs and CRP concentrations associated with single infections of *E. canis*, *B. canis*, and *H. canis*.

## Materials and Methods

### Ethical approval

The research protocol was approved by the Institutional Review Board of Chulalongkorn University Animal Committee (approval no. 1931052). All methods were performed in accordance with relevant guidelines and regulations.

### Study period and location

Blood samples were collected from Bangkok and its vicinity area during January-December 2020.

### Sample collection

A total of 650 canine (*Canis familiaris*) blood samples were collected and examined for health status and blood parasite infection. Some cases showed clinical signs of blood parasite infection, including anorexia, lethargy, pale mucous membranes, and fever. Only a few cases exhibited jaundice. Samples were collected in EDTA tubes and serum collection tubes and suspected blood parasite infections were examined by buffy coat thin blood smear and confirmed by polymerase chain reaction according to Rucksaken *et al*. [8]. The numbers of dogs with *B. canis*, *E. canis*, or *H. canis* single infections were 13, 20, and 14, respectively. The criteria for normality were healthy dogs with no clinical signs of blood parasite infection and no history of ectoparasite infestation. Blood chemistry profiles of normal dogs were in the normal range [12]. All serum samples were kept at −20°C until analysis.

### Serum protein profile determination and electrophoresis

Serum from normal (n=9), *B. canis* (n=13), *E. canis* (n=20), and *H. canis* (n=14) single infections was analyzed for total serum protein and SPEPs. Total serum protein was measured by the Biuret colorimetric test (Human^®^, Wiesbaden, Germany). The serum protein samples were separated by agarose gel electrophoresis (SPIFE^®^ split beta SPE kit; Helena Laboratories, TX, USA). Fifteen microliters (1.3 mg) of the serum protein sample were placed in each well, and electrophoresis was performed at 400 V for 6 min. The gel was pre-dried at 53°C for 12 min, stained with acid blue staining solution, and destained in citric acid destaining solution. All steps were carried out in an automated machine (Spife^®^ 3000; Helena Laboratories). The density of each serum protein band in the electrophoresis pattern was measured and analyzed using QuickScan Touch (Helena Laboratories).

### Measurements of CRP concentration

CRP concentrations of blood parasite-positive serum samples were measured by fluorescent immunoassay (Vcheck Canine CRP 2.0 Test kit; Bionote, South Korea). About 5 μL of each sample was diluted in 4 mL diluent buffer from the test kit. About 100 μL of the diluted sample was mixed and added to the test device. The CRP concentration was displayed on the screen after 5 min. A CRP concentration above 30 mg/L was considered abnormal.

### Statistical analysis

SPEP data for blood parasite single infections were tested using analysis of variance. Tukey’s test was used for pairwise comparisons between single infections and normal dogs. Significance was set at p<0.05. The normal distribution of SPEP data was tested using GraphPad Software, (GraphPad, San Diego, CA, USA).

## Results

**Figure-1:**
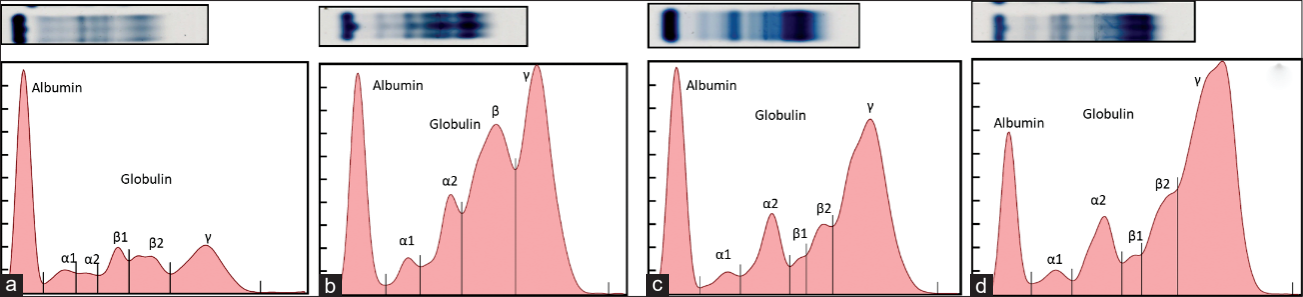
Representations of the serum electrophoresis patterns of normal dogs (A) and dogs with single infection with *Ehrlichia canis* (B), *Babesia canis* (C), and *Hepatozoon canis* (D).

SPEPs were determined by agarose gel electrophoresis (Figure-1). Tables-1 and 2 show the total protein, albumin, and globulin fractions and A/G ratios in single blood parasite infections. Comparisons were made among single infections of *B. canis*, *E. canis*, or *H. canis* in normal dogs and between groups. There were no significant differences among the three groups of blood parasite infections. In this study, the monoclonal gammopathy was 39% (5 of 13) of *B. canis* and 35% (7 of 20) of *E. canis* single infections. In dogs with *H. canis* single infection, the serum protein pattern showed β-γ bridging and an increase in β- and γ-globulin peaks in 43% (6 of 14) of the cases.

**Table-1:** The results of relative concentration of serum protein fraction (%) and albumin/globulin (A/G) ratio in normal dogs and dogs infected with *Babesia canis, Ehrlichia canis*, or *Hepatozoon canis* single infections (mean±SD).

Variables	Normal (n=9)	*Babesia canis* (n=13)	*Ehrlichia canis* (n=20)	*Hepatozoon canis* (n=14)	p-value
Albumin	42.43±6.29	32.38±9.29*	27.18±7.84*	23.15±11.64*	<0.05
α1-globulin	6.02±1.86	5.85±2.96	5.19±1.80	3.80±1.72*	<0.05
α2-globulin	6.40±3.95	8.35±5.62	10.87±5.78*	6.74±5.42	<0.05
β1-globulin	10.40±4.47	10.92±5.30	10.20±5.72	10.54±6.81	n.s.
β2-globulin	13.90±4.30	18.89±14.10*	20.81±8.23*	20.11±9.07*	<0.05
γ-globulin	25.32±8.71	23.57±13.25	25.93±9.87	33.89±10.43*	<0.05
A/G ratio	0.74±0.19	0.51±0.20*	0.39±0.17*	0.38±0.23*	<0.05

p-value–significance of the analysis of variance; n.s.=Not significant.

* Significant difference between normal and *Babesia canis, Ehrlichia canis*, or *Hepatozoon canis* at p<0.05

**Table-2:** The results of total protein and absolute values of protein fraction (g/L) in normal dogs and dogs infected with *Babesia canis, Ehrlichia canis*, or *Hepatozoon canis* single infections (mean±SD).

Variables (g/L)	Normal (n=9)	*Babesia canis* (n=13)	*Ehrlichia canis* (n=20)	*Hepatozoon canis* (n=14)	p-value
Total protein	88.47±7.26	68.25±16.46	86.19±22.26*	96.67±30.65*	<0.05
Albumin	24.22±9.72	25.08±8.34	22.85±6.63	22.29±4.70	n.s.
α1-globulin	3.78±2.44	4.08±1.80	4.40±2.04	3.57±1.83	n.s.
α2-globulin	4.00±3.84	6.31±4.71	9.40±5.88*	7.00±6.85	<0.05
β1-globulin	6.33±4.66	8.00±4.14	8.65±4.99	10.79±8.27	n.s.
β2-globulin	12.00±3.61	13.43±12.14*	17.90±8.44*	21.64±13.60*	<0.05
γ-globulin	13.67±5.32	18.62±13.55	23.00±12.17*	34.86±17.58*	<0.05

p-value – significance of the analysis of variance; n.s.=Not significant.

* Significant difference between normal and *Babesia canis, Ehrlichia canis*, or *Hepatozoon canis* at p<0.05

CRP concentrations were detected by fluorescent immunoassay. CRP concentrations in dogs infected with *B. canis* (n=6) were between 52.0 and 200.0 mg/L, with a mean of 127.62 mg/L. In dogs infected with *E. canis* (n=6), CRP concentrations were between 50.2 and 166.7 mg/L, with a mean of 105.35 mg/L. In dogs infected with *H. canis* (n=6), CRP concentrations were between 10.0 and 51.2 mg/L, with a mean of 45.6 mg/L. Dogs had increased CRP concentration, so their health status had to be observed during the treatment method.

## Discussion

Serum protein profile is one of the standard tests used to monitor health and disease status, such as infections and acute and chronic inflammatory responses. In this study, the average relative concentration of serum protein fraction albumin levels and A/G ratios significantly decreased, whereas the absolute total protein of *H. canis* significantly increased. Decreased albumin concentrations are usually caused by acute inflammation, liver damage, starvation or cachexia, digestive disorders, or kidney diseases [13]. However, the pathogenesis of *B. canis*, *H. canis*, and *E. canis* infections is usually caused by acute inflammation and/or liver damage [9,14]. Decreased A/G ratios result from a reduction in albumin concentrations and an increase in globulin concentrations. In the previous report, ehrlichiosis (in subclinical and chronic diseases) was associated with significant hypoalbuminemia, hyperglobulinemia, and hypergammaglobulinemia [15]. The serum protein profile of *B. canis*-infected dogs showed decreased albumin concentrations and A/G ratios but increased a- and β-globulin concentrations. The abnormalities in β-globulin concentrations might be due to hypertransferrinemia [16,17]. The total protein of *H. canis* increased, probably due to hyperglobulinemia caused by the stimulation of the humoral response of the organism to severe chronic inflammation [18,19].

For the globulin fraction, the relative concentrations and absolute values of β2-globulin protein levels significantly increased in *B. canis*, *H. canis*, and *E. canis* infections. An increased β2-globulin level may result from increased C3a (complement) protein concentrations. Complements are involved in the regulation of inflammatory processes, and this complement protein plays a role in the development of intravascular hemolysis, especially in babesiosis [16,17]. The γ-globulin fractions increased in all three groups, with significantly increased absolute protein values in *E. canis* and *H. canis* infections. The γ-globulin fraction consists of various classes of immunoglobulins, and an increase in the fraction could lead to monoclonal gammopathy (narrow peak) or polyclonal gammopathy (broad peak). Monoclonal gammopathies result from a single line of B lymphocytes or plasma cells, whereas polyclonal gammopathies are usually an indication of chronic inflammation and chronic liver damage [13]. In this study, monoclonal gammopathy was found in 35% of *E. canis* and 39% of *B. canis* single infections. In a previous study, polyclonal and benign monoclonal gammopathies were observed in *E. canis*-infected dogs [20]. In dogs with *H. canis* single infection, the serum protein pattern showed β-γ bridging and an increase in the β- and γ-globulin peaks in 43% of the cases. The β-γ bridging pattern is usually found in liver damage [21]. The γ-globulin concentrations of *H. canis* single infection differed significantly from other blood parasite infections because hepatozoonosis has a longer life cycle than others and produces a chronic immune response [14].

CRP is a major acute-phase protein in dogs, mostly synthesized in the liver after tissue damage caused by infection, inflammation, or trauma. The acute-phase response is an innate host defense mechanism during tissue injury or immunological disorders and in the early stage of blood parasite infections. It is responsible for the accumulation and activation of granulocytes and mononuclear cells, releasing cytokines, interleukin (IL)-1, IL-16, and tumor necrosis factor-a [22]. In this study, the average CRP concentrations in dogs with *E. canis*, *B. canis*, and *H. canis* single infections were higher than the reference range (>30 mg/L). The average CRP concentration in *B. canis* single infection was higher than *E. canis* and *H. canis* single infections. In canine hepatozoonosis, most dogs were within normal levels, and a few increased. In a previous study, the CRP concentration in dogs infected with *E. canis* increased during the acute stage of infection; this increase could eliminate *E. canis* in macrophages of infected dogs. The CRP level might help assess the severity of inflammatory damage in *E. canis-* infected dogs, and veterinarians may use this information to choose inflammatory therapy [23]. The typical hematological abnormality of blood parasite infections, including ehrlichiosis, babesiosis, and hepatozoonosis, is anemia. Ehrlichiosis and babesiosis might be caused by canine immune-mediated anemia (IMHA) [24,25]. Serum CRP concentrations in canine autoimmune hemolytic anemia and primary IMHA are increased [26,27].

## Conclusion

SPEPs in a blood parasite single infection mostly showed decreased albumin levels and A/G ratios and increased β2- and γ-globulin levels. CRP concentrations extremely increased in dogs with all blood parasite infections. SPEPs and CRP levels can help veterinarians monitor health status and blood parasite problems in sick dogs during the treatment process.

## Authors’ Contributions

SA and PT: Designed the study, corrected data, data analysis and interpretation, and drafted the manuscript. The authors have read and approved the final manuscript.
